# Assessment of pyloric sphincter physiology after Ivor-Lewis esophagectomy using an endoluminal functional lumen imaging probe

**DOI:** 10.1007/s00464-022-09714-9

**Published:** 2022-12-01

**Authors:** Stefanie Brunner, Florian Lorenz, Thomas Dratsch, Lorenz Schröder, Ulrich Toex, Benjamin Babic, Hans Friedrich Fuchs, Thomas Schmidt, Christiane Josephine Bruns, Tobias Goeser, Seung-Hun Chon

**Affiliations:** 1 Interdisciplinary Endoscopy Unit, Department of General, Visceral, Cancer and Transplantat Surgery, University Hospital of Cologne, Kerpener Street 62, 50937 Cologne, Germany; 2 Interdisciplinary Endoscopy Unit, Department of Gastroenterology and Hepatology, University Hospital of Cologne, Cologne, Germany; 3 Department of Diagnostic and Interventional Radiology, University Hospital of Cologne, Cologne, Germany; 4 University of Cologne, Cologne, Germany

**Keywords:** EndoFlip™, Pyloric distensibility, Esophagectomy, Delayed gastric conduit emptying, Gastroparesis

## Abstract

**Objective of the study:**

The most common functional complication after Ivor-Lewis esophagectomy is the delayed emptying of the gastric conduit (DGCE) for which several diagnostic tools are available, e.g. chest X-ray, upper esophagogastroduodenoscopy (EGD) and water-soluble contrast radiogram. However, none of these diagnostic tools evaluate the pylorus itself. Our study demonstrates the successful measurement of pyloric distensibility in patients with DGCE after esophagectomy and in those without it.

**Methods and procedures:**

Between May 2021 and October 2021, we performed a retrospective single-centre study of all patients who had an oncological Ivor-Lewis esophagectomy and underwent our post-surgery follow-up programme with surveillance endoscopies and computed tomography scans. EndoFlip™ was used to perform measurements of the pylorus under endoscopic control, and distensibility was measured at 40 ml, 45 ml and 50 ml balloon filling.

**Results:**

We included 70 patients, and EndoFlip™ measurement was feasible in all patients. Successful application of EndoFlip™ was achieved in all interventions (*n* = 70, 100%). 51 patients showed a normal postoperative course, whereas 19 patients suffered from DGCE. Distensibility proved to be smaller in patients with symptoms of DGCE compared to asymptomatic patients. For 40 ml, 45 ml and 50 ml, the mean distensibility was 6.4 vs 10.1, 5.7 vs 7.9 and 4.5 vs 6.3 mm^2^/mmHg. The differences were significant for all three balloon fillings. No severe EndoFlip™ treatment-related adverse events occurred.

**Conclusion:**

Measurement with EndoFlip™ is a safe and technically feasible endoscopic option for measuring the distensibility of the pylorus. Our study shows that the distensibility in asymptomatic patients after esophagectomy is significantly higher than that in patients suffering from DGCE. However, more studies need to be conducted to demonstrate the general use of EndoFlip™ measurement of the pylorus after esophagectomy.

In the field of surgery, pyloric function plays a crucial role in the postoperative recovery after esophagectomy [1]. The most common functional complication, occurring in around one third of the patients, is delayed emptying of the gastric conduit (DGCE).

Around 14% of the affected patients are treated with an endoscopic intervention [2]. DGCE is associated with a reduced quality of life, increased morbidity and prolonged hospitalization [3]. In addition, DGCE can lead to a series of adverse events such as pneumonia, anastomotic leakage, and prolonged ICU and hospital stay [4]. The pathophysiology is not fully understood. However, the obligatory transsection of vagal nerves and the altered anatomy of the gastric conduit are suspected to be contributing causes [5]. There are several diagnostic tools available: e.g. anamnesis, chest X-ray, upper esophagogastroduodenoscopy (EGD), and water-soluble contrast radiogram. However, none of these diagnostic tools evaluate the pylorus itself.

One novel endoscopic approach to evaluate the pylorus is an endoscopic functional luminal imaging probe (EndoFlip™, Medtronic, Minneapolis, USA). It measures pressure, diameter and distensibility (DI) in order to study biomechanical properties of gastrointestinal sphincters [6]. In the past years, several studies have been performed to implement EndoFlip™ measurement for the pylorus [7, 8].

However, only few available studies investigate the efficacy of EndoFlip™ measurement in the pylorus after esophagectomy. The purpose of this study is to focus on the pyloric distensibility in a patient cohort after standardized oncological esophagectomy. Our goal was to evaluate the pyloric distensibility in asymptomatic postoperative patients and compare it to patients suffering from DGCE after upper gastrointestinal tract surgery.

## Materials and methods

### Study setting and patients

This retrospective study was conducted at the Interdisciplinary Endoscopy Unit (Head Department of Gastroenterology and Hepatology) at the University Hospital Cologne, a European high-volume centre for tumour entities of the upper gastrointestinal tract. Data were retrieved from our prospectively maintained endoscopic database “Clinic WinData” (version 8.06; E&L medical system GmbH, Erlangen, Germany) and from our hospital database “Orbis” (version 08,043,101; Agfa HealthCare N.V., Belgium). The following information was collected: demographic and clinical patient characteristics, details of the disease, surgical outcome data, and endoscopic findings.

Between May 2021 and October 2021, 70 patients underwent Endoflip™ measurement. We included all postoperative patients after oncological Ivor-Lewis esophagectomy who underwent our follow-up programme with surveillance endoscopies and computed tomography scans. In our standardized follow-up programme, and endoscopy is performed 6, 12, 18 and 24 month after surgery. All patients under the age of 18 were excluded as well as patients who did not give their informed consent.

All patients underwent a standardized interview about symptoms and quality of life (Patient Assessment of Upper Gastrointestinal (PAGI) Symptoms and Quality of Life).

DGCE was defined according to the scoring system for late DGCE [9]. Delayed contrast passage on upper GI water-soluble contrast radiogram or timed barium swallow at least 14 days after surgery was defined as DGCE when combined with characteristic symptoms such as early satiety, nausea and vomiting.

### EndoFlip™

The EndoFlip™ 2.0 Impedance Planimetry System (EF-200, Medtronic, Minneapolis, USA) is connected to a measurement balloon catheter (EF-325 N) of 2.8 mm diameter and 8 cm length with 16 impedance electrodes, which can be in- and deflated with 0 ml to 50 ml of saline solution in 1 ml steps, using an integrated pump. Pressure inside the balloon (in mm of mercury, mmHg) as well as diameter of the balloon (in mm) at the location of the 16 electrodes are measured and recorded on a local memory stick continuously during examination. Analysis of these parameters allows the simulation of the sphincteric region as a three-dimensional real-time profile. The balloon catheter is calibrated at atmospheric pressure before placement. Distensibility is calculated by dividing a cross-sectional area in mm^2^ by pressure in mmHg. EndoFlip™ measures impedance planimetry with real-time images [6].

### EndoFlip™ measurement

The requirement for EGD with EndoFlip™ is a fasting period of at least 6 h for food and non-clear liquids and 2 h for clear liquids. Two investigators and two assisting nurses were present during EGD. The patient was positioned in left lateral recovery position. EndoFlip™ was then placed under endoscopic guidance with a flexible video esophagogastroduodenoscope (e.g. GIF-H190; GIF-XP180N; Olympus Medical Systems, Tokyo, Japan) with photo and video documentation. All procedures were performed under sedation with propofol (e.g. Fresenius Kabi Germany GmbH).

In our study, we first assessed the esophagus as well as the gastric conduit for pathologies such as anastomotic leak or ischemic conduit. Pylorus or duodenum was initially not intubated in order to prevent any mechanical impact on pyloric distensibility.

The complete EndoFlip™ catheter (EF-325 N) was lubricated with gel and inserted orally alongside the endoscope. The correct catheter positioning was continuously confirmed through endoscopic vision. Correct positioning was also verified with the shape of the pylorus represented as a narrow segment with a decreased cross-sectional area (CSA) on the screen. The balloon was inserted throughout the pylorus, positioning the pylorus in the middle of the balloon (presented in image 1).

After correct and stable positioning, the EndoFlip™ was initially filled with 30 ml saline solution and then in additional 5 ml steps up to a final 50 ml (presented in image 2a-c). After every 5 ml step, a 30 s intermission was taken to allow measurement. Afterwards, the balloon was deflated in 5 ml steps with intermissions of 30 s after every deflation. Finally, after completing EndoFlip™ measurements, the catheter was fully deflated and removed.

### EndoFlip™ analysis

During the whole examination, the pressure, smallest diameter and the smallest CSA were continuously recorded. The minimum distensibility was calculated as CSA/pressure for every filling volume. A cut-off of 10 mm2/mmHg at 40 ml of inflation was determined in previous studies of healthy subjects [1]. Using this threshold value of 10 mm2/mmHg, pyloric distensibility was classified as either normal (> 10 mm2/mmHg) or decreased (< 10 mm2/mmHg) among subjects.

### Gastroparesis Cardinal Symptom Index Score (GCSI), Patient Assessment of Upper Gastrointestinal—Symptoms (PAGI-Sym) and Quality of Life (PAGI-QoL)

The GCSI consists of three subscales of the PAGI-SYM, selected to measure important symptoms related to gastroparesis: nausea/vomiting (three items: nausea, retching, and vomiting), post-prandial fullness/early satiety (four items: stomach fullness, inability to finish a normal sized meal, feeling excessively full after meals, and loss of appetite), and bloating (two items: bloating and belly visibly larger). The GCSI total score is constructed as the average of the three symptom subscales [10]. It can range from 0 to 5, with higher scores reflecting greater symptom severity.

The PAGI-SYM contains 20 items measuring six domains: nausea/vomiting (three items); post-prandial fullness/early satiety (four items); bloating (two items); upper abdominal pain (two items); lower abdominal pain (two items) and heartburn/regurgitation (seven items). Patients rated the severity of each symptom on a 6-point Likert scale from 0 (none) to 5 (very severe) [11].

The PAGI-QoL contains 30 items covering five subscales: Daily Activities (ten items), Clothing (two items), Diet and Food Habits (seven items), Relationships (three items) and Psychological Well-Being (eight items). Patients rated the severity of each symptom on a 6-point Likert scale from 0 (none) to 5 (very severe). Subscale scores are calculated by averaging across the items within the specific subscale after reversing item scores. The range of scores is 0–5; higher scores indicate a better quality of life [12].

### Data collection and statistical methods

Data were collected retrospectively, including but not limited to age, gender, body mass index, endoscopic findings, date of the operation, distensibility, PAGI-QoL score and UICC stadium. Continuous variables are presented as means and range. Categorical data are presented as numbers and percentages. The student T test, for continuous variables, and Chi square test, for nominal or categorical variables, were used for all bivariate analyses. All tests were two sided, with statistical significance set at *P* ≤ 0.05. Data were analysed by the Stata 11.0 (StataCorp, College Station, TX), SPSS Statistics Version 28 (IBM Corp., Armonk, NY, USA) for Windows (Microsoft Corp, Redmond, WA) and Microsoft Excel Version 2013 for Windows (Microsoft Corp, Redmond, WA).

#### Study outcome

Our primary endpoint is to distinguish the pyloric distensibility in asymptomatic patients after esophagectomy in comparison to the distensibility of healthy volunteers described in the literature. Moreover, we aim to distinguish the pyloric distensibility of asymptomatic postoperative patients from patients suffering from DGCE in order to better select patients suffering from DGCE for a therapeutic pyloric intervention. Our secondary outcomes are treatment-related events and feasibility of EndoFLIP™ after esophagectomy.

### Approval

The manuscript was submitted to the local ethics committee, which stated that we are exempt from applying for ethical approval as, under German law no separate ethics application and statement of ethical approval by the local ethics committee is required for performing purely retrospective clinical studies.

## Results

### Baseline demographics and procedural characteristics

70 subjects were analysed between May 2021 and October 2021. The analysed patients had a median age of 64 (28–83) years. The patients’ characteristics were similar between the two groups as shown in Table 1. Food retention during endoscopy and patients with UICC stadium III occurred more often in DGCE patients.

**Table 1:** Patients’ characteristics in the two different groups

	DGCE	Non DGCE
*N*	19	51
Age (mean)	62 (35–80)	63 (28–83)
Men/women	14/5	43/8
UICC stadium		
Stadium 0 (*n*) (percentage)	2 (10%)	8 (16%)
Stadium I	6 (32%)	17 (33%)
Stadium II	4 (21%)	15 (30%)
Stadium III	7 (37%)	10 (19%)
Stadium IV	0 (0%)	1 (2%)
Smoking status (active or former)	26% (*N* = 5)	35% (*N* = 18)
BMI (kg/m^2^) (mean)	26 (17, 7–31, 4)	26 (18, 4–37, 8)
ASA (mean) [95% KI]	2 [1, 85–2, 15]	2 [1, 87–2, 13]
CCI (mean) [95% KI]	0,26 [0, 12–0, 4]	0,35 [0,19–0,51]
Retention of food in the endoscopy	58% (*N* = 11)	41% (*N* = 21)

**Fig. 1:**
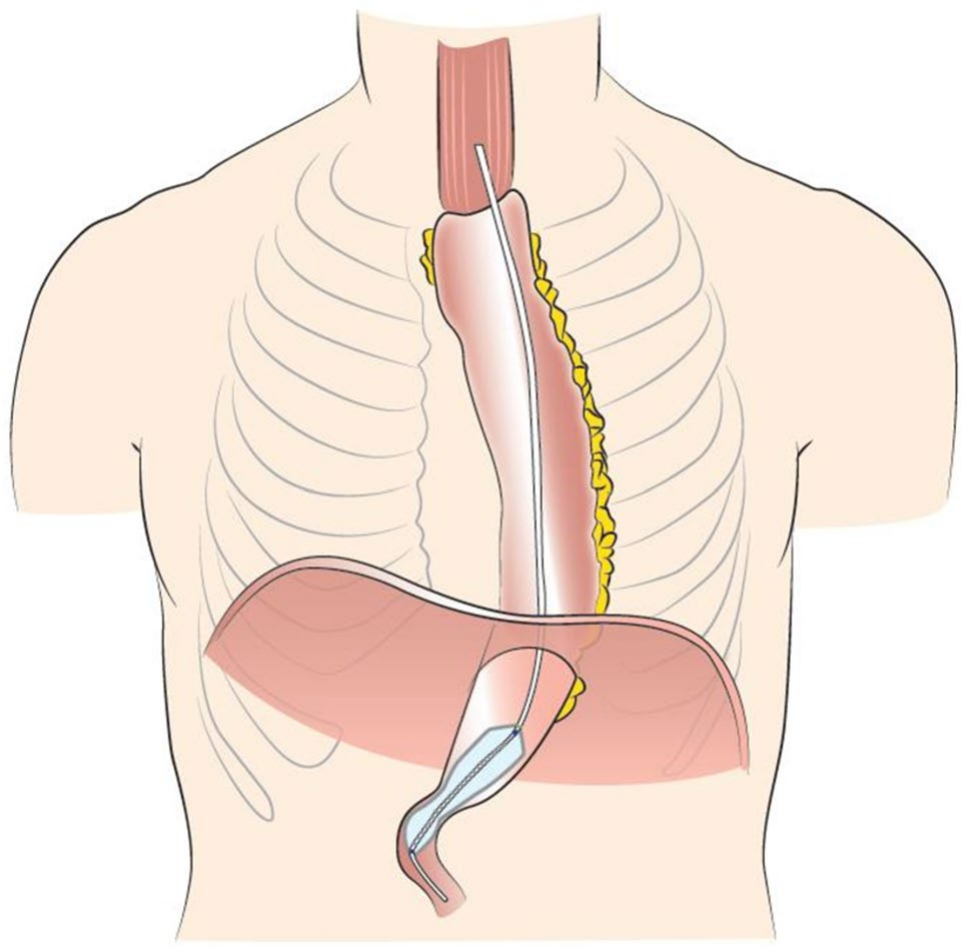
Placement of EndoFlip™ throughout the pylorus

**Fig. 2:**
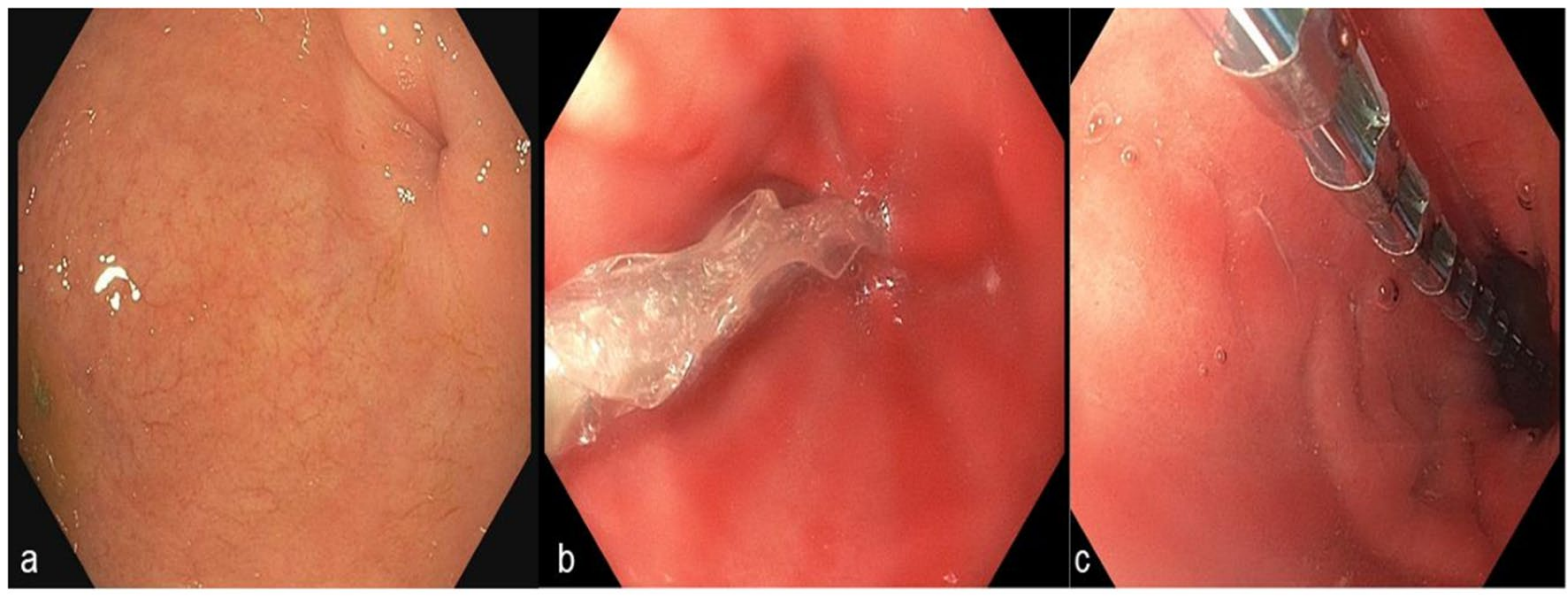
**a** Endoscopic image showing the pylorus before EndoFlip™ measurement. **b** Implantation of EndoFlip™ catheter (EF-325 N). **c** Endoscopic view of inflated EndoFlip™ catheter and pylorus

In all 70 patients, we achieved technical success in all interventions (*n* = 70, 100%). 51 patients presented no signs of DGCE, while 19 patients presented signs of it. No patient received a prior endoscopic or surgical pyloric intervention: e.g. pyloric dilatation or Botox injection (Figs. 1, 2).

### GCSI, PAGI-SYM, and PAGI-QoL

**Fig. 3:**
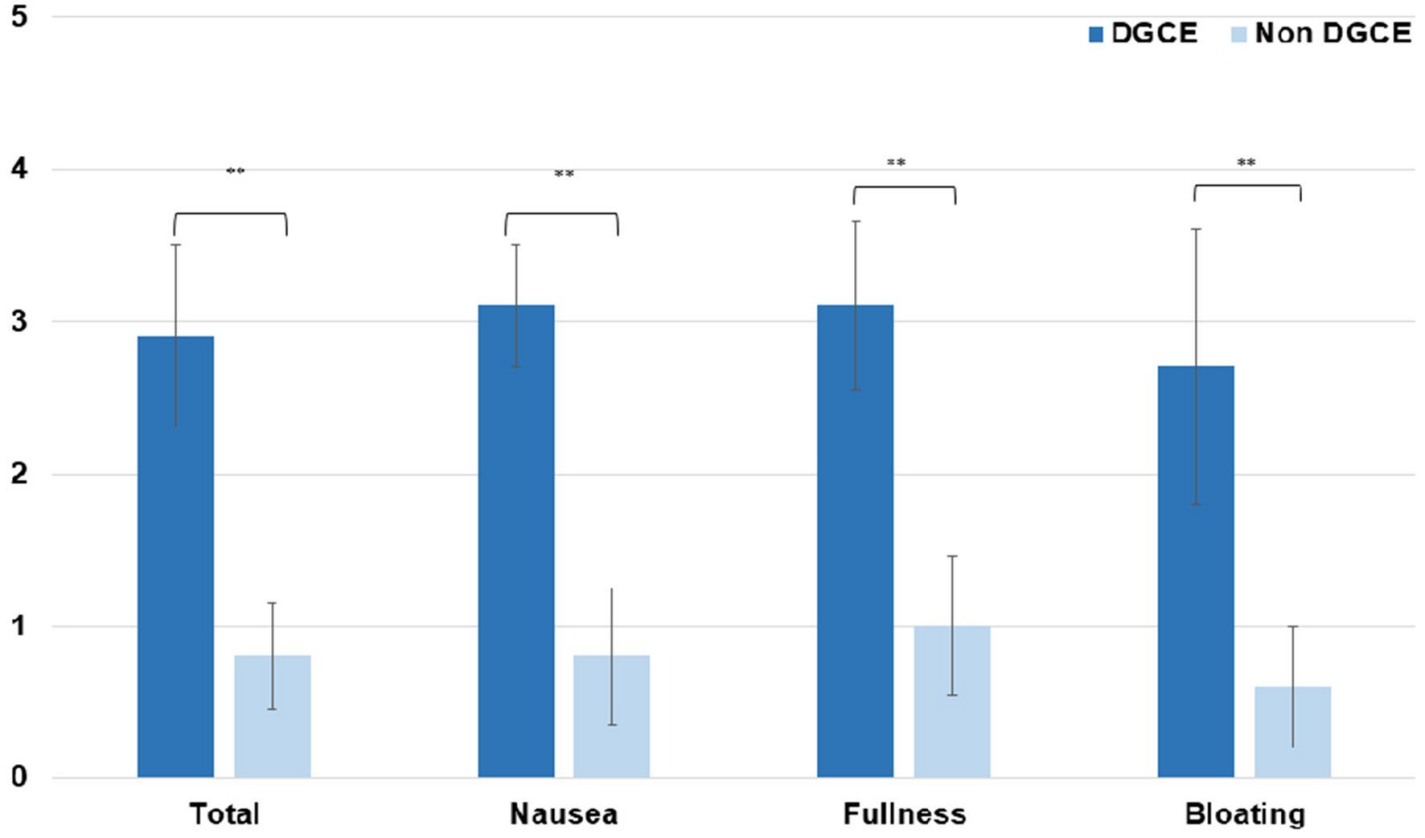
GCSI scores of DGCE and asymptomatic patients, **P < .05

**Fig. 4:**
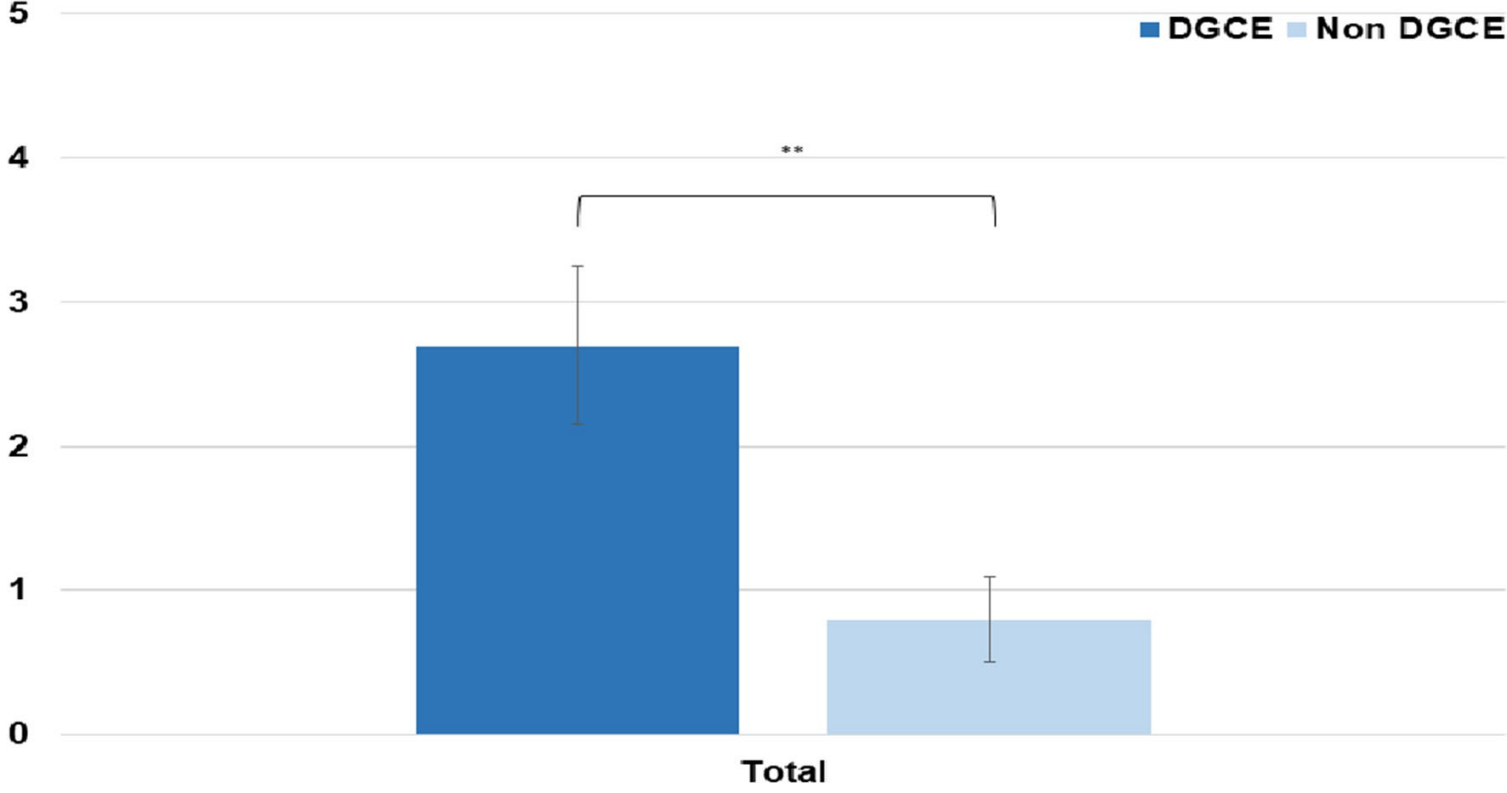
PAGI-SYM scores of DGCE and asymptomatic patients, **P < .05

**Fig. 5:**
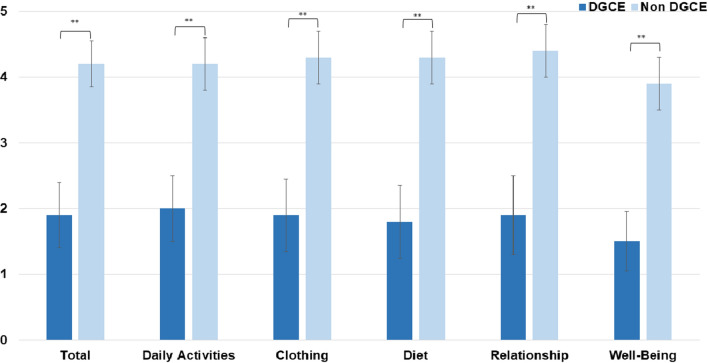
PAGI-QoL scores of DGCE and asymptomatic patients, **P < .05

Data on the GCSI score, PAGI-Sym score and PAGI-QoL score were available in 19 patients with DGCE symptoms and in 51 patients without DGCE symptoms. As expected, the PAGI-Sym and GCSI scores were significantly reduced in the DGCE group in comparison to asymptomatic patients (Fig. 3 and 4) whereas PAGI-QoL showed a significantly higher score in patients without DGCE (Table 2, Fig. 5).

**Table 2:** Presenting GCSI, PAGI-SYM, and PAGI-QoL in DGCE and non-DGCE patients

	DGCE	Non DGCE
GCSI total (mean) [SD]	2.9 (1.2)	0.8 (0.7)
GCSI nausea (mean) [SD]	3.1 (0.8)	0.8 (0.9)
GCSI post prandial fullness (mean) [SD]	3.1 (1.1)	1.0 (0.9)
GCSI bloating (mean) [SD]	2.7 (1.8)	0.6 (0.8)
PAGI SYM total (mean) [SD]	2.7 (1.1)	0.8 (0.6)
PAGI QoL total (mean) [SD]	1.9 (1.0)	4.2 (0.7)
PAGI QoL daily activities (mean) [SD]	2.0 (1.0)	4.2 (0.8)
PAGI QoL clothing (mean) [SD]	1.9 (1.1)	4.3 (0.8)
PAGI QoL diet (mean) [SD]	1.8 (1.1)	4.3 (0.8)
PAGI QoL relationship (mean) [SD]	1.9 (1.2)	4.4 (0.8)
PAGI QoL psychological well-being (mean) [SD]	1.5 (0.9)	3.9 (0.8)

### EndoFlip™ measurements (pyloric distensibility)

All our patients showed a reduced distensibility after surgery, with mean measurements of 10.1 mm^2^/mmHg at 40 ml and lower in all balloon fillings.

**Fig. 6:**
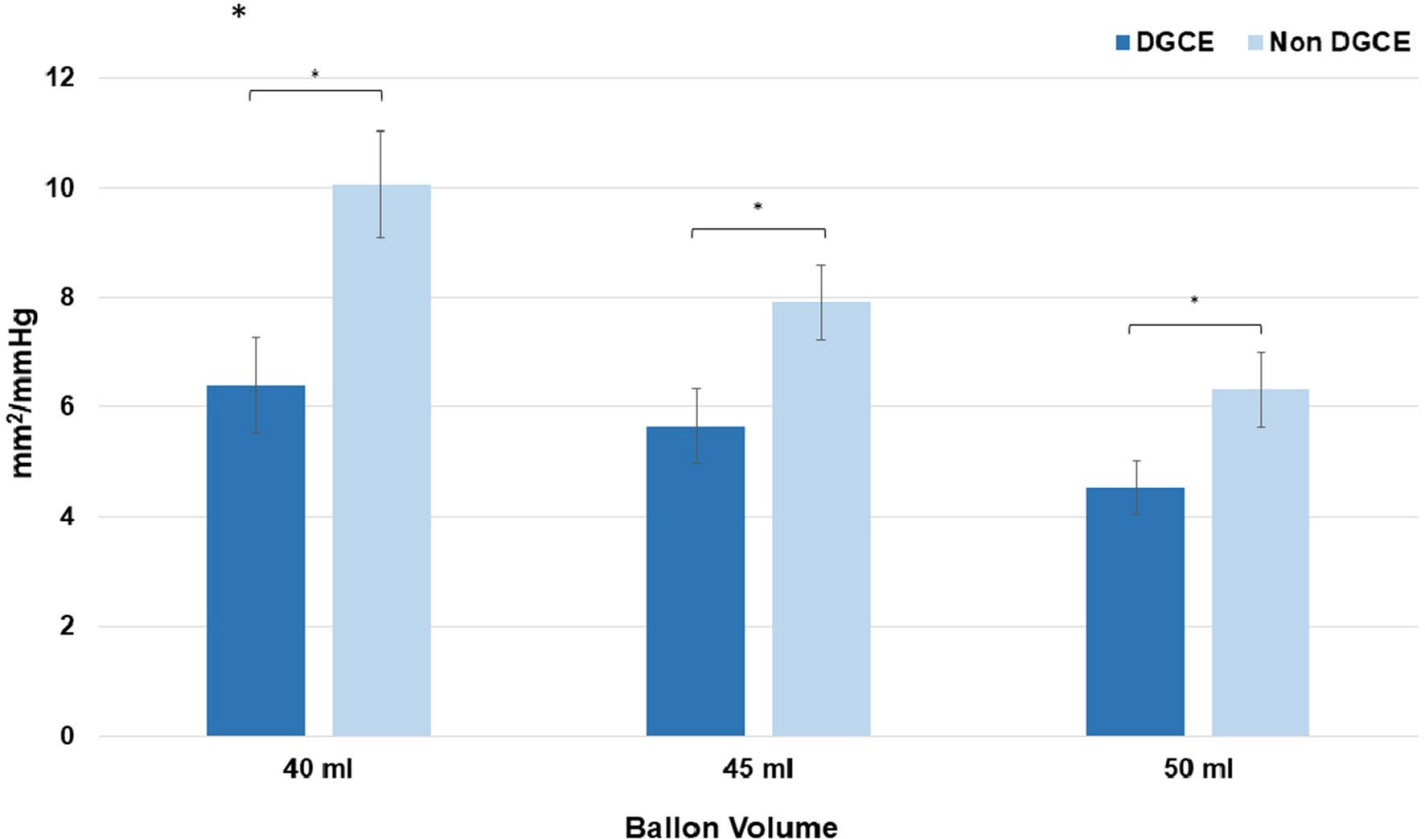
Pyloric sphincter distensibility with EndoFlip™ balloon inflated at 40, 45, and 50 ml of DGCE and asymptomatic patients, *P < .05

In patients with signs of DGCE, the mean distensibility was significantly lower in all balloon fillings compared to those in asymptomatic post-surgical patients (Table 3 and Fig. 6). The highest significant difference in distensibility was measured at 40 ml balloon filling, proving to be the most reliable filling level.

**Table 3:** Pyloric sphincter distensibility with EndoFlip™ balloon inflated at 40, 45, and 50 ml of DGCE and asymptomatic patients,* p* < .05

	Ballon Volume		
	40 ml	45 ml	50 ml
DGCE (mm^2^/mmHg) [SD]	6.4 (2.8)	5.7 (2.2)	4.5 (1.6)
Non-DGCE (mm^2^/mmHg) [SD]	10.1 (4.2)	7.9 (3.0)	6.3 (3.0)

Significances were overall clearer for distensibility levels in comparison to diameter and pressure values (data not shown). Consequently, distensibility seems to be the most reliable parameter.

**Fig. 7:**
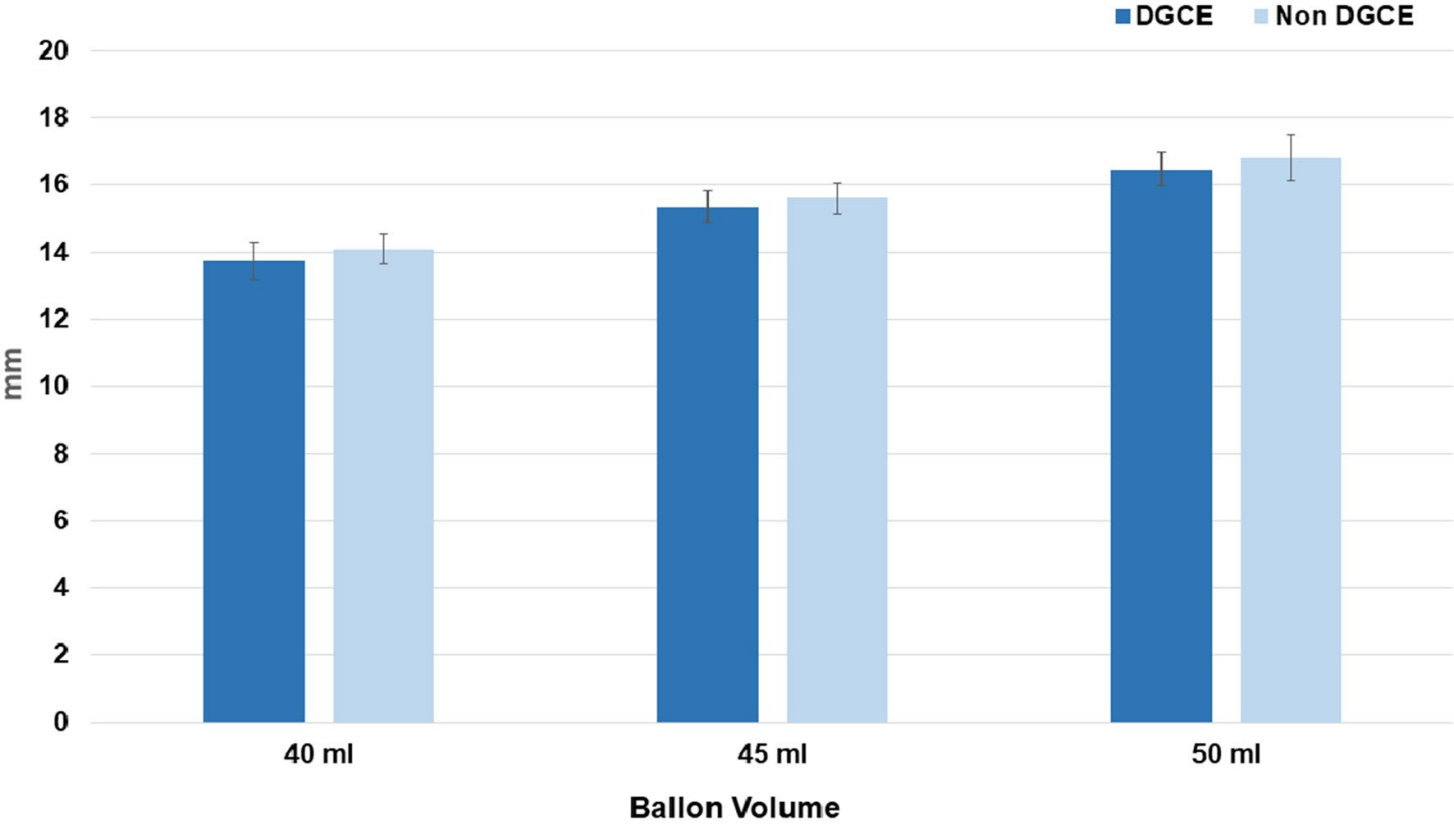
Pyloric sphincter diameter with EndoFlip™ balloon inflated at 40, 45, and 50 ml of DGCE and asymptomatic patients, *P* > .05

With regard to diameter, there were no significant differences between DGCE and asymptomatic patients (Fig. 7, Table 4).

**Table 4:** Pyloric sphincter diameter with EndoFlip™ balloon inflated at 40, 45, and 50 ml of DGCE and asymptomatic patients, *p > .05

	Ballon Volume		
	40 ml	45 ml	50 ml
DGCE (mm) [SD]	13.7 (1.8)	15.3 (1.5)	16.5 (1.6)
Non-DGCE (mm) [SD]	14.1 (1.9)	15.6 (2.0)	16.8 (2.1)

No severe EndoFlip™ treatment-related adverse events occurred, and no patient died because of the endoscopic intervention.

## Discussion

Delayed emptying of the gastric conduit after Ivor-Lewis esophagectomy is a severe complication and has a negative impact on patients’ postoperative outcome. A novel innovative therapeutic and diagnostic approach in this context is the use of EndoFlip™ to evaluate the function of the pylorus. It is an established endoscopic diagnostic option of the lower esophageal sphincter—especially in achalasia.

However, evidence of the effectiveness of EndoFlip™ as a diagnostic tool for the pylorus is limited. So far, only few studies have investigated the use of EndoFlip™ and published small sample sizes [13]. Our aim with this study was an evaluation of the normal postoperative pyloric physiology in comparison to the postoperative gastroparetic pylorus using EndoFlip™.

Recent studies have shown the feasibility and safety of EndoFlip™ in a surgical setting [14]. However, there is a lack of standardization among these studies in terms of probe placement and inflation protocol [15]. In our study, we committed ourselves to an exact and standardized measurement. First of all, we did not intubate the pylorus before measurement to avoid inaccurate data. Moreover, the EndoFlip™ balloon was placed under endoscopic vision and continuously monitored. Additionally, a 30 s time span between measurements was ensured in order to receive stable data.

Our study found a decreased pyloric distensibility at 40 ml, 45 ml and 50 ml of inflation in asymptomatic patients after esophagectomy in comparison to that in healthy volunteers. Desprez et. al. describe the pyloric distensibility of 25 mm^2^/mmHg at 40 ml balloon inflation in healthy volunteers, while the normal lower range was set at 10 mm^2^/mmHg [16]. Using the threshold of 10 mm2/mmHg, altered pyloric distensibility with a mean of 10.1 mm2/mmHg at 40 ml was found in our patients who experienced a normal postoperative course—a result that is confirmed by earlier studies[16]. Interestingly it seems that the pure transsection of the vagal nerve alters the pyloric distensibility, but without necessarily causing symptoms.

DGCE is the most common functional challenge after esophagectomy [2] [17]. Previous studies show that symptoms such as vomiting and nausea can lead to a decreased pyloric distensibility [18]. With regard to patients suffering from DGCE, we demonstrated a significantly decreased distensibility in comparison to a postoperatively altered pylorus. Especially the highest filling volume showed a significantly decreased distensibility in symptomatic patients. In our study, we measured distensibility levels lower than 7 in patients suffering from DGCE. Consequently, we set the threshold that differentiated DGCE from a normal postoperative course at 7 mm2/mmHg and 40/45 ml balloon filling in our EndoFlip™ measurement. A larger patient cohort is needed to verify that this is an appropriate threshold to choose in order to safely diagnose DGCE with this diagnostic modality.

Building on our results, we want to better select patients suffering from DGCE for pyloric intervention, such as pyloric dilatation. Recent studies have focused on the influence of systematic pyloric intervention during esophagectomy [19]. They could demonstrate that systemic pyloric intervention such as pyloromyotomy, pyloroplasty and Botox injection could not reduce the occurrence of DGCE. However, adverse events like dumping syndrome or biliary reflux increased significantly. Therefore, we need an improved diagnostic tool of selecting postoperative patients for pyloric interventions. In our study, EndoFlip™ has enabled us to distinguish the normal postoperative pyloric distensibility from pylorospasm. This is in line with previous reports which suggest that pyloric distensibility can predict patient outcome after Gastric Per-Oral Endoscopic Pyloromyotomy (G-POEM) [7]. Moreover, no serious adverse event in the form of intestinal perforation occurred after EndoFlip™ treatment.

Another important aspect of our study is the evaluation of life quality impacting our patients with dyspeptic symptoms. By measurement with the PAGI-QoL score, we were able to show that patients in the DGCE group have a significantly reduced quality of life in comparison to asymptomatic patients. These results are also echoed in similar studies focusing on gastroparesis and patients’ life quality [20].

The strength of this study is its inclusion of a homogenous group of patients who all underwent a standardized oncological Ivor-Lewis esophagectomy due to esophageal cancer. In addition, most studies so far have only included a small number of patients [16]. We investigated a homogenous patient cohort of 70 patients in total, thereof 19 patients suffering from DGCE and 51 patients experiencing a normal postoperative course.

Limitations of this study are the retrospective design and the missing evaluation of healthy volunteers. Previous studies have defined standard values for pyloric distensibility in healthy volunteers; hence, we decided to use these existing data.

## Conclusion

Our study shows that measurement with EndoFlip™ is safe and feasible after oncological Ivor-Lewis esophagectomy. Our results suggest that pyloric distensibility is significantly decreased in patients after esophagectomy suffering from DGCE in comparison to asymptomatic patients. The use of EndoFlip™ might help to diagnose DGCE at an early stage and may be useful to target patients for pyloric dilatation. Further evaluation and identification of selection criteria for this method are strongly recommended.
